# Efficacy validation of a low-cost handmade simulator (SIMCA-COW) in palpation, ultrasonography evaluation, and artificial insemination in cows

**DOI:** 10.14202/vetworld.2023.144-148

**Published:** 2023-01-23

**Authors:** Carolina Balão da Silva, Elvira Matilla Pinto

**Affiliations:** 1 Agrarian School of Elvas, Polytechnic Institute of Portalegre, Elvas, Portugal;; 2 VALORIZA– Research Centre of Endogenous Resource Valorization, Portalegre, Portugal

**Keywords:** artificial insemination, bovine rectal palpation, simulator, ultrasonographic evaluation

## Abstract

**Background and Aim:**

Using simulators in high education enables practical training by repetition in circumstances close to reality reducing the stress of both animal and operator. The limited resources of veterinary schools, the increase in the number of students in lecture halls, and the low availability of animals for teaching due to welfare regulations, reduce teaching opportunities with live animals being simulator as the better alternative. This study aimed to assess the efficacy of a low-cost handmade simulator (SIMCA-COW) in eight veterinary students inexperienced in palpation, ultrasonography evaluation, and artificial insemination in cows.

**Materials and Methods:**

Two sets of exercises were carried out: structure localization by rectal palpation and ultrasonography were evaluated by the inexpert veterinary students in the SIMCA-COW simulator. Also, evaluated the length of time to insert the insemination catheter through the cervix in the inert simulator during four sessions.

**Results:**

All the students were able to palpate both structures and to locate the body and both uterine horns by ultrasonography. Cervix and follicles were located by 5/8 (62.5%) students and 3/8 (37.5%) students found the corpus luteum by ultrasonography. A reduction in time span between the first and fourth intrauterine insemination attempts was observed (8.26 ± 2.7 vs. 3.69 ± 1.7; mean ± standard error; p < 0.05).

**Conclusion:**

The simulator validated in this study (SIMCA-COW) allows training and learning by repetition, saving the limitations found in live animal practice.

## Introduction

Bovine rectal palpation is commonly used for reproductive tract evaluation and the most popular among large animal veterinarians [1]. Rectal palpation makes it possible to evaluate the structures of the feminine reproductive system (cervix, uterus, uterine horns, and ovaries) and perform gestation diagnosis. Pregnancy can be determined by rectal ultrasound [2] or by manual detection of definitive signs, including the allantocorium membrane, amniotic vesicle, placentomes, or fetus [3]. These reproductive techniques usually require practice on a minimum of 200 cows to achieve a constant level of experience in palpation and structure identification [4]. Artificial insemination in cows is a technique that requires skill and dexterity because the insemination catheter with semen must pass through three cartilaginous rings that cows have in the cervix. The procedure consists of introducing a gloved hand in the rectum to hold the cervix and using the other hand to guide the insemination catheter through the cervix [5].

Training and teaching programs are fundamental for the acquisition of skills with direct application of this technique [6]. Animal welfare is one of the priorities of the third Strategic Plan of the World Organization for Animal Health [7]. There is controversy in the literature regarding the negative effects of students practicing on cattle. Some authors claim that teaching rectal palpation is a practice that induces a stress response in cows, as well as a reduction in vagal tone [8] and that repetitive rectal palpation and insemination practices can affect bovine health and productivity [9]. Other studies suggest the safety of using live animals for bovine palpation training [10]. The use of simulators in high education enables practical training by repetition in similar to real-life situations, ensuring the correct performance of the technique before applying it to live animals [11]. At present, there are numerous real-size simulators and models of different materials that allow veterinary students training to perform basic learning procedures [12]. Several studies have evaluated the use of simulators to improve the learning process of bovine rectal palpation [4, 13–15].

This study aimed to assess the usefulness of a low-cost handmade simulator called SIMCA-COW as a model for structure recognition training through rectal palpation, ultrasound evaluation, and intrauterine insemination by inexperienced veterinary students.

## Materials and Methods

### Ethical approval

The nature of the study did not require ethical approval because slaughterhouse viscera was used to develop the simulator.

### Study period and location

The study was conducted from June 2022 to July 2022 in the Agrarian School of Elvas from Polytechnic Institute of Portalegre (Portugal). Inexperienced veterinary volunteer students were from different Veterinary Universities in Portugal.

### Inert animal

**Figure-1:**
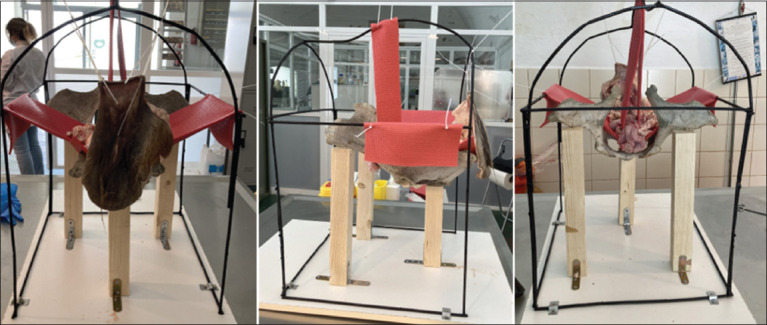
Left: Back view of SIMCA-COW simulator shows vulva and anus of the cow; Centre: lateral view shows the pelvis and the support straps; Right: The frontal view shows hanging horns and ovaries from the cow uterus.

The simulator called SIMCA-COW was handmade using the pelvis of a cow anchored to a rigid support to which a metal structure was added. Straps were placed on the hip bones to support the viscera, making the model anatomically similar to a real cow. The abattoir-sourced wombs were extracted with the vulva, anus, and part of the rectum, allowing to perform techniques as they would be done similar to *in vivo* (Figure-1). The external structures of the simulator covered the entire reproductive apparatus except for the vulva and the anus, thereby forcing the students to identify the structures by palpation and not by sight. The front of the simulator could be uncovered so the teacher could check and guide students throughout the procedures. The total price for the construction of the simulator did not exceed 40$. The SIMCA-COW simulator was used to test the efficacy in two training exercises for veterinary students.

### Experimental design

In this study, two sets of exercises were carried out: (1) In the first exercise, inexperienced students with interest in the bovine species were given some basic theoretical knowledge and afterward performed a simulator test to recognize the structures. The theoretical training was based on reviewing the anatomy of the reproductive system of cows, the estrus cycle, physiological alterations, and ultrasound images. For the practical simulator test, the initially stood at the exposed side of the simulator and guided each student in recognition of the reproductive structures. Subsequently, the students practiced the acquired knowledge by themselves in the same reproductive organ and an individual exam was made to assess structure identification by each student. Afterward, an ultrasonograph was provided to each individual without prior practical training in order to analyze the skills acquired by structure recognition made in the previous exercise (Table-1). To increase the echogenicity of the structures, the rectilinear probe was inserted on a palpation glove filled with ultrasound gel and tied to prevent gel leakages. (2) The second exercise measured the time taken to pass the catheter insemination through the cervix, with each student repeating the procedure four times (Table-2).

**Table-1:** Reproductive cow parts found by inexpert veterinary students by rectal palpation in the simulator.

Students	Cervix	Uterine corpus	Right horn	Left horn	Right ovary	Left ovary	Follicles	Corpus luteum
n = 8	8/8 (100%)	8/8 (100%)	8/8 (100%)	8/8 (100%)	8/8 (100%)	8/8 (100%)	8/8 (100%)	8/8 (100%)

**Table-2:** Reproductive cow structures found by inexpert veterinary students by ultrasonography evaluation in the simulator.

Students	Cervix	Uterine corpus	Right horn	Left horn	Right ovary	Left ovary
n = 8	5/8 (62.5%)	8/8 (100%)	8/8 (100%)	8/8 (100%)	5/8 (62.5%)	3/8 (37.5%)

### Statistical analysis

The data were analyzed using the statistical software package Sigma Plot Version 12.3 for Windows (Systat Software, Chicago, IL, USA). The means of the values were represented with standard error (mean ± standard error of the mean). The groups were compared with a one-way analysis of variance to check whether they followed a Gaussian distribution. Differences between values were considered significant when p < 0.05 according to Tukey´s honestly significant difference test.

## Results

In the first exercise, all the students managed to find the cervix, uterine body, both uterine horns, and ovaries through rectal palpation in the simulator after training with the teacher (Table-1). Using the ultrasonography, all of the students were able to locate the body and both uterine horns; five students (62.5%) successfully located the cervix and ovarian structures (follicles), and only three students (37.5%) located the corpus luteum (Table-2).

**Figure-2:**
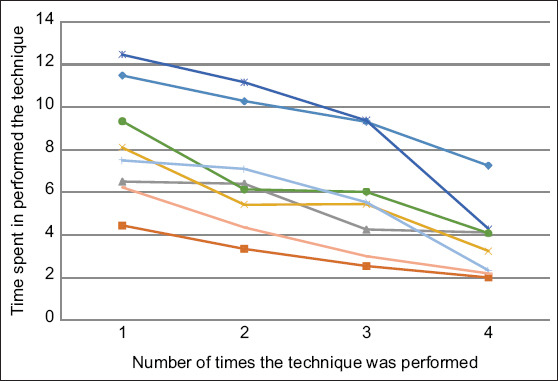
Time spent in the training of artificial insemination technique by inexpert student in the simulator. Colorful lines represent each student. The points represent the number of times the technique was performed by students.

**Figure-3:**
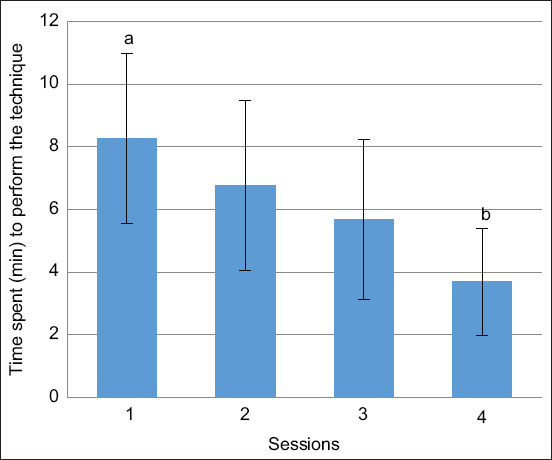
Mean of time spent by students divided in each try. Bars represent the mean with the standard error of the mean. Significant differences were found between the first and fourth try and are represented by letters a and b (p < 0.05).

Over the four attempts of the second exercise, a decrease was observed in the time spent by all individuals in performing the intrauterine artificial insemination technique (Figure-2). Significant differences (p < 0.05) were observed between the time averages of the students for the first artificial insemination attempt and the fourth and final attempt (8.26 ± 2.7 vs. 3.69 ± 1.7) (Figure-3).

## Discussion

This study aimed to test the efficacy of a handmade simulator (SIMCA-COW) made at a low-cost. Simulation in the practice of veterinary medicine represents a safe environment where students’ errors can be quickly corrected [16]. Moreover, simulation learning in inert models provides greater self-confidence in the process of rectal palpation with animals *in vivo* [17]. The low-cost handmade simulator analyzed in this study (SIMCA-COW) seems to provide, like other simulators on the market, personalized teaching and a specific evaluation of the technique. Although each individual’s innate psychomotor skills affect learning time, development and improvement of cognitive and motor skills [18], teaching strategies are constructed in terms of repetitive time according to each curriculum, and tasks are objectively assigned to improve the quality of this methodology [19].

There is controversy among authors about how rectal palpation affects pregnant cows. Some studies suggest that rectal palpation in early stages may be a major cause of pregnancy loss [20, 21], while others provide evidence that palpation does not affect fetal loss [10, 22] although it may be a stressor [8]. This fact, combined with the limited resources of veterinary schools, the increase in the number of students in lecture halls and the low availability of animals for teaching due to welfare regulations, reduces teaching opportunities in live animals [23], justifying the increased use of simulators in theriogenology.

The eight students who participated in this study were able to locate by palpation all the reproductive structures with the SIMCA-COW model. The use of abattoir viscera is a procedure widely used in veterinary practices and their use progressively reduces the amount of time needed for the students to find the structures [24]. The development of reproductive management techniques that combine ultrasound with new and existing reproductive technologies improves reproductive practices in cattle [25]. With the SIMCA-COW and after prior tuition by the professor, all of the students managed to find the body and the uterine horns by palpation and five students (62.5%) were able to locate the cervix and presented ultrasound images of ovarian follicles. However, only three students (37.5%) managed to present the ultrasound image of the corpus luteum. Although ultrasound imaging is more accurate than rectal palpation to asses reproductive organs, it is difficult to distinguish between the development and regression of the corpus luteum [26]. The development of educational programs to train students in the use of ultrasonography for routine reproductive examination is a critical step toward the rapid implementation of this technology in the dairy industry [25].

The majority of large animal simulators are focused on bovine rectal palpation and/or pregnancy diagnosis [11], while intrauterine insemination in cows usually requires extensive practice in live animals. One study indicated that in more than 50% of the cases analyzed, the veterinarian was not sufficiently trained to deposit semen in the uterine body, leading to frequent intracervical insemination and, therefore, a decrease in fertility [27]. It is essential to train students in these reproductive techniques and offer the opportunity to perform the technique in inert models to reduce the stress of both animal and operator. In 2015, Nagel *et al*. [28] found that the cortisol levels and heart rate of students performing ultrasound examination in a simulator were lower than those who performed it directly on living animals. Simulators as educational tools can help students to overcome their anxiety while practicing some procedures [29], improving their learning curve.

Recently, simulator use in veterinary education has increased significantly, allowing consistent, practical teaching and reducing the number of live animals used [30]. The low-cost SIMCA-COW handmade simulator evaluated in this study seems to be useful in the repetitive training of students, showing a decrease in the time spent on the artificial insemination technique. Another application of the handmade simulator tested in this study was to help the professor to train students in palpation, ultrasonographic evaluation, and artificial insemination in cows. The use of simulation models in veterinary medicine is on the rise. A recent study analyzed the results and time of different veterinary simulation techniques showing evident effectiveness in increasing clinical skills with simulator training programs [31]. Moreover, it is of utmost importance to develop tools such as simulators to help educators to adhere to modern ethical guidelines that aim to protect animal welfare and reduce the need to obtain live animals solely for teaching or pedagogic research purposes [11].

## Conclusion

The SIMCA-COW low-cost handmade simulator, aimed at safe student practices in the area of veterinary medicine, seems to be an alternative to simulators of different materials available on the market at higher costs, allowing for repetition training and solving limitations of *in vivo* practicing.

## Authors’ Contributions

EMP: Conceived the idea and built the simulator. EMP and CBS: Collected the data, performed the computations, verified the analytical methods and prepared the manuscript. Both authors have read and approved the final manuscript.
